# Ferroptosis-related lncRNAs: Distinguishing heterogeneity of the tumour microenvironment and predicting immunotherapy response in bladder cancer

**DOI:** 10.1016/j.heliyon.2024.e32018

**Published:** 2024-05-31

**Authors:** Zhan Yang, Xiaoqi Li, Lijun Zhou, Yaxian Luo, Ning Zhan, Yifan Ye, Zhichao Liu, Xiaoting Zhang, Tao Qiu, Lining Lin, Lianjie Peng, Yiming Hu, Chaoran Pan, Mouyuan Sun, Yan Zhang

**Affiliations:** a Department of Urology, The First Affiliated Hospital of Wenzhou Medical University, Wenzhou, 325000, China; b Stomatology Hospital, School of Stomatology, Zhejiang University School of Medicine, Zhejiang Provincial Clinical Research Center for Oral Diseases, Key Laboratory of Oral Biomedical Research of Zhejiang Province, Cancer Center of Zhejiang University, Engineering Research Center of Oral Biomaterials and Devices of Zhejiang Province, Hangzhou, 310000, China

## Abstract

Ferroptosis, a cell death pathway dependent on iron, has been shown in research to play a role in the development, advancement, and outlook of tumours through ferroptosis-related lncRNAs (FRLRs). However, the value of the FRLRs in bladder cancer (BLCA) has not been thoroughly investigated. This research project involved developing a predictive model using ten specific FRLRs (AC099850.4, AL731567.1, AL133415.1, AC021321.1, SPAG5-AS1, HMGA2-AS1, RBMS3-AS3, AC006160.1, AL583785.1, and AL662844.4) through univariate COX and LASSO regression techniques. The validation of this signature as a standalone predictor was confirmed in a group of 65 patients from the urology bladder tumour database at the First Affiliated Hospital of Wenzhou Medical University in Wenzhou, China. Patients were categorized based on their median risk score into either a low-risk group or a high-risk group. Enrichment analysis identified possible molecular mechanisms that could explain the variations in clinical outcomes observed in high-risk and low-risk groups. Moreover, we explored the correlation between FLPS and immunotherapy-related indicators. The ability of FLPS to forecast the effectiveness of immunotherapy was validated by the elevated levels of immune checkpoint genes (PD-L1, CTLA4, and PD-1) in the group at high risk. We also screened the crucial FRLR (HMGA2-AS1) through congruent expression and prognostic conditions and established a ceRNA network, indicating that HMGA2-AS1 may affect epithelial-mesenchymal transition by modulating the Wnt signalling pathway through the ceRNA mechanism. We identified the top five mRNAs (NFIB, NEGR1, JAZF1, JCAD, and ESM1) based on random forest algorithm and analysed the relationship between HMGA2-AS1, the top five mRNAs, and immunotherapy, and their interactions with drug sensitivities. Our results suggest that patients with BLCA have a greater sensitivity to four drugs (dasatinib, pazopanib, erismodegib and olaparib). Our study provides new insights into the TME, key signalling pathways, genome, and potential therapeutic targets of BLCA, with future guidance for immunotherapy and targeted precision drugs.

## Highlights

• Construction of a clinically instructive ferroptosis-related lncRNA prognostic signature (FLPS).
• Create a competing endogenous RNA network to uncover the potential molecular pathways of BLCA.
• Offer a fresh citation for the treatment of BLCA using immunotherapy and medication. We showed that patients with BLCA are sensitive to four drugs (dasatinib, pazopanib, erismodegib and olaparib).

## Graphical abstract

BLCA is the most prevalent and highly lethal malignancy that affects the urinary system, and patients with BLCA often have poor prognoses. Ferroptosis is crucial in regulating metabolism and redox biology as the elicitation of programmed cancer cell death. As a result, the activation of ferroptosis has become a hopeful strategy for treating cancer, particularly in the field of tumour immunotherapy.**Construction and validation of FLPS**: The central ferroptosis-related genes (FRGs) were constructed and validated. Then, ten key ferroptosis-related lncRNAs (FRLRs) were screened using Spearman, univariate COX, and LASSO regression analyses. A prognostic signature called FLPS, related to ferroptosis and constructed from these lncRNAs, was developed to assess their prognostic value for BLCA patients using univariate and multivariate Cox regression analyses. The predictive accuracy of FLPS was confirmed in both the TCGA database and a local cohort.**FLPS in predicting Immunotherapy's Efficacy:** Based on the relative coefficient-weighted expression levels of these ten key FRLRs, a risk score was calculated for all patients. Immune checkpoint proteins (PD-L1, CTLA4, and PD-1) showed high expression levels in the populations at high risk. FLPS has the potential to predict the effectiveness of immunotherapy, and it is believed that immune checkpoint inhibitors could be useful in treating BLCA.**Construction and Drug sensitivity of the ceRNA network**:Biological function and enrichment analysis of FLPS showed that FLPS is closely related to the tumour microenvironment (TME). We screened a vital lncRNA (HMGA2-AS1), established a ceRNA network, and identified one microRNA (miRNA) node and seven mRNA nodes. Subsequently, by performing drug sensitivity analysis on the ceRNA network, we screened for dasatinib and pazopanib, which are sensitive to HMGA2-AS1, and olaparib and erismodegib, which are sensitive to ESM1, and these targeting drugs could potentially be applied to the treatment of BLCA.

**Figure:**
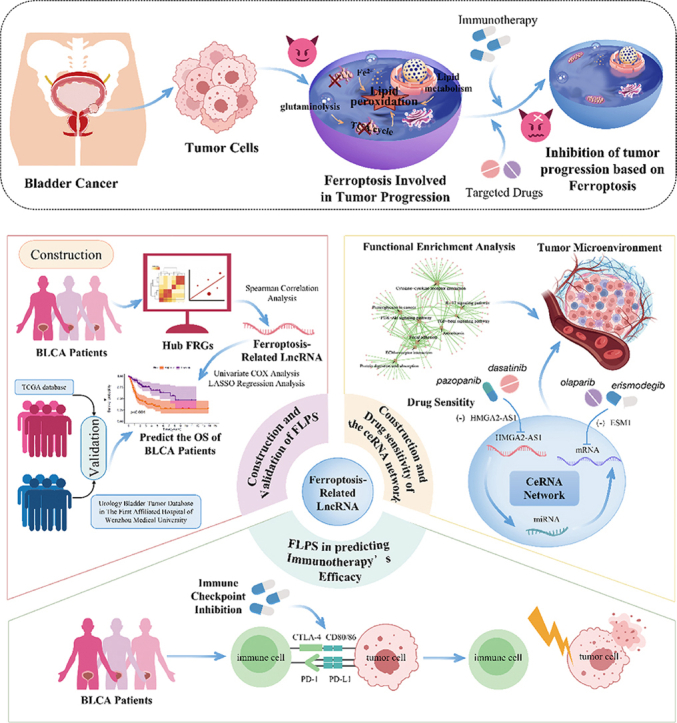

## 1 Introduction

Bladder cancer (BLCA) is the most prevalent and deadly form of cancer affecting the urinary system [1]. BLCA is ranked as the tenth most common malignant tumour because of its high rates of metastasis, recurrence, complications and death [2]. Novel treatments such as adjuvant chemotherapy, targeted drugs and immune checkpoint inhibition are improving results for patients with advanced BLCA [3,4]. Regrettably, the outlook for individuals diagnosed with BLCA is frequently grim, as the 5-year survival rate stands at a mere 15 % [5]. Identifying high-risk patients and implementing appropriate personalized treatments are challenging tasks because of the complicated pathological process of BLCA, which encompasses tumour transformation, immune cell dysfunction, and molecular heterogeneity [6]. Some biomarkers have demonstrated prognostic value in relation to patients. For example, response to cisplatin-based neoadjuvant chemotherapy can be predicted by serum vascular endothelial growth factor and DNA damage repair gene defects, including ERCC2, ATM, RB1 and FANCC [7,8]. Insufficient biomarkers exist for predicting the response to immunotherapy, necessitating a deeper comprehension of the molecular characteristics and pathological mechanisms of tumour development. The discovery of new prognostic biomarkers can guide future research and improve clinical outcomes and treatment approaches for BLCA.

Ferroptosis is a new form of cell death that relies on iron and lipid reactive oxygen species (ROS) build up, rather than apoptosis, to regulate metabolism and redox biology and induce programmed cancer cell death [9]. Thus, the initiation of ferroptosis has become a hopeful treatment method, especially in cancer immunotherapy [10,11]. Emerging BLCA studies have confirmed that ferroptosis involves numerous tumour-associated signalling pathways in the tumour microenvironment (TME), including immune system reprogramming [12]. Targeting key pathways of ferroptosis in combination with immunotherapy may provide a new therapeutic strategy for the treatment of BLCA.

Long non-coding RNAs (lncRNAs), which are transcripts exceeding 200 nucleotides in length, have the ability to control immune reactions and are essential in BLCA tumor formation, metabolism, advancement, and spread [13,14]. Recently, Cheng et al. reported that lncRNA SNHG1 facilitates tumour proliferation and represses apoptosis by regulating PPARγ ubiquitination in BLCA [15]. Moreover, Chen et al. demonstrated that exosomal lncRNA LNMAT2 promotes lymphatic metastasis in BLCA [16]. Furthermore, lncRNA TNRC6C-AS1 has been reported to be B cell-specific and correlated with the immunosuppressive phenotype and cancer immunotherapy [17]. Several studies have investigated the association between long noncoding RNAs (lncRNAs) and ferroptosis. In gastric cancer, hypoxia-triggered lncRNA-CBSLR controls ferroptosis by affecting CBS through the m6A-YTHDF2 pathway [18]. The long non-coding RNA H19 competes with miR-19b-3p, enhancing the transcriptional activity of its natural target, ferritin heavy chain 1 (FTH1), which is a marker for ferroptosis [19]. Although previous studies focused on lncRNAs and ferroptosis, the concrete functions of ferroptosis-related lncRNAs (FRLRs) in BLCA progression and prognosis remain unclear. Moreover, studies have not examined the combined predictive ability of multiple FRLRs and the effectiveness of the corresponding immunotherapy in patients with BLCA. The ceRNA network functions as an intricate regulatory system after transcription, enabling lncRNAs, mRNAs, and other RNAs to vie for microRNAs (miRNAs) through the sharing of a common miRNA response element (MRE) as a natural miRNA sponge. These lncRNAs act as ceRNAs to regulate mRNA expression and modulate protein levels, leading to tumourigenesis and cancer progression. Creating a ceRNA network can enhance comprehension of the potential biological pathways through which lncRNA plays a role in BLCA.

This study validates the significance of ferroptosis-associated genes (FRGs) and FRLRs in BLCA. Following this, a new prognostic signature called FLPS was developed to forecast the outcome of BLCA patients based on ferroptosis-related lncRNA. A group of patients from the urological bladder tumour database at the First Affiliated Hospital of Wenzhou Medical University in Wenzhou, China, was utilized to confirm the precision of this signature. This prognostic signature opens up a new perspective for the investigation of potential molecular prognostic markers and therapeutic targets in patients with BLCA. Furthermore, we conducted functional enrichment analysis to investigate the underlying mechanisms, aiding clinicians in gaining a deeper understanding of the pathological features and development of BLCA. The key signature-FRLR was identified, and a ceRNA network was implemented to detect potential molecular mechanisms. Ultimately, we explored drug sensitivity and immunotherapy analysis of the ceRNA network, which may provide a unique and promising therapeutic approach for BLCA immunotherapy and precision therapy.

## 2 Results

### 2.1 Exploring the relationship between the ferroptosis-related genes and cell population in BLCA

**Fig. 1:**
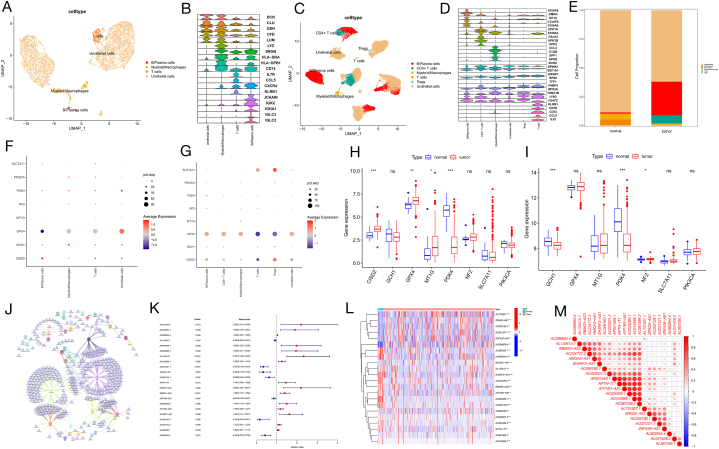
Screening and identification of ferroptosis-related lncRNAs **(A)** All four cell populations in normal tissues were annotated with “singleR” and cell marker genes. **(B)** Violin plot shows the expression level of the top five DEGs of each cell population in normal tissues. **(C)** All six cell populations in tumour tissues were annotated with “singleR” and cell marker genes. **(D)** Violin plot shows the expression level of the top five DEGs of each cell population in tumour tissues. **(E)** Comparison of cell population of normal and tumour tissues **(F)** Dot plot shows the expression levels of these eight central FRGs in each cell population in normal tissues. **(G)** Dot plot shows the expression levels of these eight central FRGs in each cell population in tumour tissues. **(H)** Differences in ferroptosis marker between normal and tumour tissues in the GSE13507 cohort. **(I)** Differences in ferroptosis marker between normal and tumour tissues in the TCGA cohort. **(J)** Co-expression network of 38 ferroptosis genes and 392 relevant lncRNAs. **(K)** Forest map of these 22 prognostic FRLRs. **(L)** Heatmap of the 22 identified prognostic FRLRs; red indicates high expression and blue indicates low expression. **(M)** Spearman's rank correlation analysis of these 22 prognostic FRLRs.

Based on the "singleR" package and previous studies, the core cells of normal tissues were annotated into four cell populations: B/plasma cells, myeloid/macrophages, T cells, and uroepithelial cells (Fig. 1A), and those of tumour tissues were annotated into six cell populations: B/plasma cells (10,641 cells), CD4^+^ T cells (2586 cells), myeloid/macrophages (517 cells), T cells (68 cells), Tregs (74 cells), and uroepithelial cells (22627 cells) (Fig. 1C). Fig. 1E shows a comparison of cell proportions between normal and tumour tissues, with a significantly higher percentage of B/plasma cells and CD4^+^ T cells in tumour tissues. Fig. 1B and D displayed the levels of expression of the initial five differentially expressed genes (DEGs) in every cell group of healthy and cancerous tissues using violin plots. Based on previous studies [20], we selected eight central FRGs that were closely related to tumour treatment from 259 FRGs, including SLC7A11, PIK3CA, PDK4, NF2, MT1G, GPX4, GCH1, and CISD2. The close relationship of these genes suggests they could have a significant impact on the development of BLCA, as shown in Supplementary Figs. S2A and B. To demonstrate the heterogeneity of ferroptosis in different cell types, we analysed the expression of central ferroptosis genes in various cell populations.

As a result, we depicted the levels of expression of these key FRGs in both normal and tumour cohorts. Tumour tissue analysis revealed the expression of each gene in different cell populations. Among them, GPX4 was upregulated in B/plasma cells, CD4^+^ T cells, and myeloid/macrophages, but downregulated in T and Treg cells (Fig. 1G), differing from FRG expression in normal tissues (Fig. 1F). In the GSE13507 and TCGA cohorts, the expression of FRGs differed between the normal and tumour groups. The findings from the GSE13507 group indicated notable variations in the levels of GCH1, PDK4, and NF2 between tumour and healthy groups, while the results from the TCGA group showed a significant contrast in the levels of CISD2, GPX4, MTG, and PDK4 between tumour and healthy tissues (Fig. 1H and I). Consequently, the disparity in FRG expression between normal and tumour tissues suggests that ferroptosis is crucial for BLCA.

### 2.2 Identification of prognostic ferroptosis-related lncRNAs in BLCA

Correlation coefficients between FRGs and lncRNAs in the TCGA cohort were calculated using the 'limma' R package. FRLRs were identified as lncRNAs with |R| > 0.6 and FDR values < 0.001, with a total of 392 lncRNAs showing correlation with ferroptosis. The study utilized correlation analysis to discover FRLRs, uncovering a relationship between 38 FRGs and 392 lncRNAs. The co-expression network can be observed in Fig. 1J. Among the 392 FRLRs, 22 were considered prognostic lncRNAs using univariate Cox regression analysis. High expression of 15 prognostic FRLRs (AL031429.2, AC022001.3, AC099850.4, AP001469.1, AC025280.1, AL133415.1, AC012568.1, NPTN-IT1, HMGA2-AS1, RBMS3-AS3, AC087286.2, ATP1B3-AS1, SH3RF3-AS1, AC234772.2, and AL583785.1) demonstrated bad prognosis of BLCA tissues, whereas seven prognostic FRLRs (AL731567.1, AC021321.1, SPAG5-AS1, AC002128.1, ZNF436-AS1, AC006160.1, and AL662844.4) exhibited good prognosis (Fig. 1K). Fig. 1L (heatmaps) shows that seven prognostic FRLRs (AL731567.1, SPAG5-AS1, AC002128.1, AC021321.1, ZNF436-AS1, AC099850.4, and HMGA2- AS1) were upregulated in BLCA tissues and 15 prognostic FRLRs (AL583785.1, AL662844.4, AL133415.1, SH3RF3-AS1, AL031429.2, RBMS3-AS3, ATP1B3- AS1, AC025280.1, AC234772.2, AC006160.1, AC022001.3, AC087286.2, NPTN-IT1, AP001469.1, and AC012568.1) were downregulated in BLCA tissues. These 22 FRLRs are closely related to each other. (Fig. 1M).

### 2.3 Development and verification of the ferroptosis-associated lncRNA predictive model in BLCA

To establish a FLPS with optimal interpretation and application efficiency for predicting OS in patients with BLCA, we randomly assigned 406 patients with BLCA to the training (204 cases) and validation (202 cases) cohorts. LASSO regression analysis (Supplementary Figs. S3A and B) was used to screen the ten key FRLRs based on the 22 prognostic FRLRs to construct the prognostic signature. Supplementary Figs. S3C–L demonstrated the importance of these ten crucial FRLRs (AC099850.4, AL731567.1, AL133415.1, AC021321.1, SPAG5-AS1, HMGA2-AS1, RBMS3-AS3, AC006160.1, AL583785.1, and AL662844.4) for BLCA patients, with ROC curves highlighting their role in BLCA development (Supplementary Fig. S4).These ten key FRLRs were highly correlated with clinical outcomes and cancer pathogenesis in patients with BLCA. We delved deeper into the connection between the ten primary FRLRs and the TME due to their strong association with the prognosis of BLCA patients. AL133415.1, HMGA2-AS1, RBMS3-AS3, AL583785.1, and AL662844.4 were positively correlated with stromal, immune, and estimated scores, whereas AL731567.1, AC021321.1, and SPAG5-AS1 were negatively correlated (Supplementary Fig. S5). With the continuous formation and growth of tumours, tumour cells gradually exhibit characteristics consistent with progenitor and stem cells, showing poor differentiation, which may contribute to immune dysfunction in cancer. We investigated the relationship between the RNA stemness score (RNAss) and the DNA stemness score (DNAss) with ten key FRLRs, which are indicators of tumour stemness. The expression levels of AL133415.1, HMGA2-AS1, RBMS3-AS3, and AL583785.1 were found to be negatively correlated with tumour stemness, as indicated by RNAss and DNAss, whereas SPAG5-AS1 was positively correlated, suggesting its potential biological function in the regulation of immune dysfunction (Supplementary Fig. S6).

Subsequently, utilizing the expression levels of these ten important FRLRs weighted by their coefficients, we computed the risk scores for every patient in both the training and validation groups by applying the specified formula:

Risk score = (0.0105 × expression of AC099850.4) + (−0.0444 × expression of AL731567.1) + (0.4875 × expression of AL133415.1) + (−0.4597 × expression of AC021321.1) + (−0.0586 × expression of SPAG5-AS1) + (0.5214 × expression of HMGA2-AS1) + (0.0206 × expression of RBMS3-AS3) + (−1.7612 × expression of AC006160.1) + (0.0287 × expression of AL583785.1) + (−0.1838 × expression of AL662844.4).

**Fig. 2:**
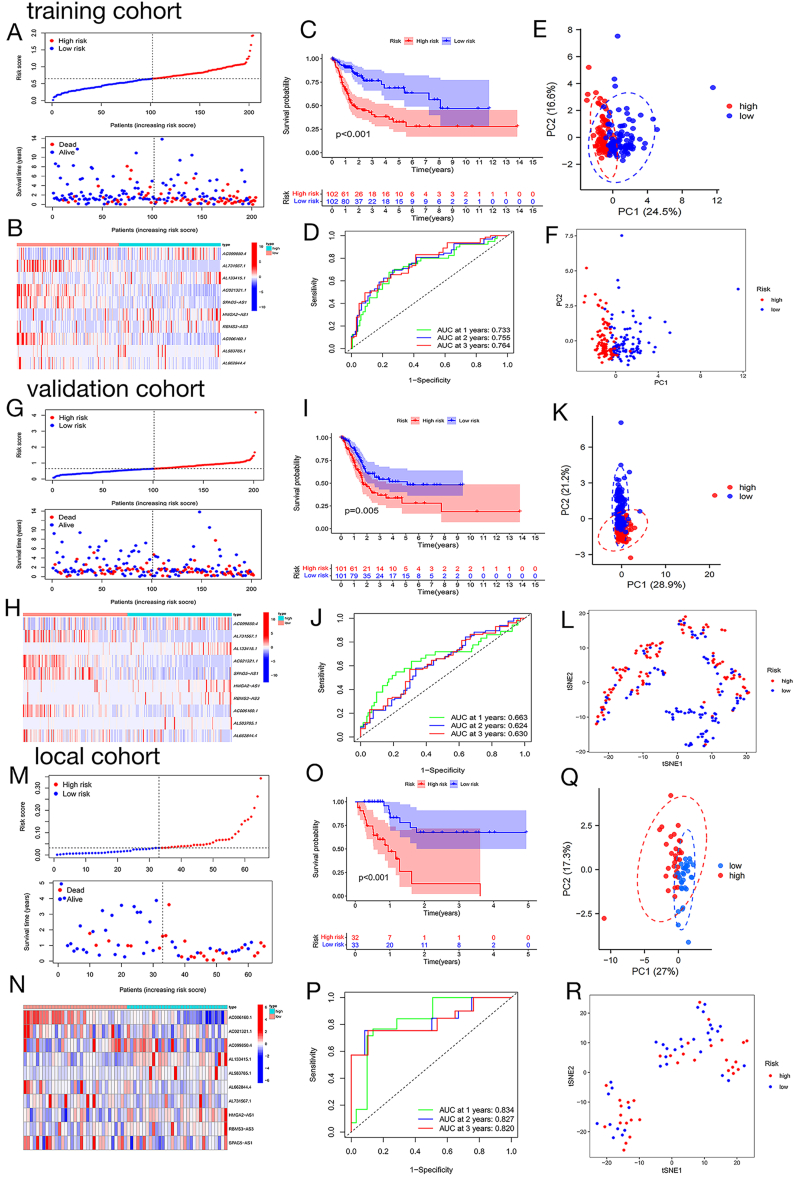
**Construction and validation of the FLPS. (A)** Distribution of risk scores and patient status for the patients with BLCA in the training cohort based on the FLPS. **(B)** Expression patterns for the signature-FRLRs in high- and low-risk groups of the training cohort. **(C)** Kaplan-Meier survival curves of OS in the high- and low-risk groups in the training cohort. **(D)** ROC curve of model predictive accuracy over time in the training cohort. **(E)** PCA of the training cohort. **(F)** t-SNE analysis of the training cohort. **(G)** Distribution of risk scores and patient status for patients with BLCA in the validation cohort based on the FLPS. **(H)** Expression patterns for the signature-FRLRs in high- and low-risk groups of the validation cohort. **(I)** K-M survival curves of OS in the high- and low-risk groups in the validation cohort. **(J)** ROC curve of model predictive accuracy over time in the validation cohort. **(K)** PCA of the validation cohort. **(L)** t-SNE analysis of the validation cohort. **(M)** Distribution of risk scores and status for the patients with BLCA in the local cohort based on the FLPS. **(N)** Expression patterns for the signature-FRLRs in high- and low-risk groups of the local cohort. **(O)** K-M survival curves of OS in the high- and low-risk groups in the local cohort. **(P)** ROC curve of model predictive accuracy over time in the local cohort. **(Q)** PCA of the local cohort. **(R)** t-SNE analysis of the local cohort.

Patients with BLCA in the training and validation cohorts were classified into low- and high-risk groups based on the median risk score. The scatter plot for survival indicated that patients in the low-risk category had a longer survival time compared to those in the high-risk category (Fig. 2A and G). Additionally, the heatmaps showed that the levels of AC099850.4, AL133415.1, HMGA2-AS1, AL583785.1, and RBMS3-AS3 were increased while AC021321.1, SPAG5−AS1, AL731567.1, AC006160.1, and AL662844.4 were decreased in the high-risk group according to Fig. 2B and H. The Kaplan-Meier survival analysis indicated that the low-risk group had a greater overall survival rate compared to the high-risk group, as illustrated in Fig. 2C and I. The ROC curves that vary with time showed AUC values of 0.733, 0.755, and 0.764 for survival at 1, 2, and 3 years in the training group, and 0.663, 0.624, and 0.630 in the validation group. Fig. 2D and J, suggesting that our signature has a better predictive ability for patients with BLCA; however, more cohorts are needed for validation. Additionally, PCA (Fig. 2E and K) and t-SNE analysis (Fig. 2F and L) showed a satisfactory distinction between high- and low-risk patient groups. Overall, our initial findings indicate that FLPS has the ability to reliably forecast the survival and outlook of individuals diagnosed with BLCA.

To further validate the accuracy of the FLPS in predicting the OS of patients with BLCA, we used a local cohort (n = 65). The local cohort was divided into two groups, designated as high-risk (n = 32) and low-risk (n = 33), based on the median risk score. Scatter plots of survival status confirmed that individuals classified as high-risk experienced shorter survival durations compared to those classified as low-risk (Fig. 2M).The heatmaps showed that AL133415.1 was specifically increased in the high-risk group, indicating its important function in BLCA advancement (Fig. 2N).The Kaplan-Meier survival analysis indicated that individuals diagnosed with bladder cancer and higher risk scores experienced poorer overall survival rates compared to those with lower risk scores (p < 0.001) as illustrated in Fig. 2O. Additionally, the local cohort achieved AUCs of 0.834 at 1 year, 0.827 at 2 years, and 0.820 at 3 years, demonstrating effective prediction of patient outcomes in BLCA cases (Fig. 2P). Additionally, the results of the PCA (Fig. 2Q) and t-SNE (Fig. 2R) analyses in the local cohort confirmed a good separation of patients with BLCA. The findings indicate that FLPS has the ability to accurately forecast patient outcomes and shows promise as a useful diagnostic indicator.

### 2.4 An independent prognostic factor for patients with BLCA was discovered in the form of a prognostic signature involving lncRNAs related to ferroptosis

**Fig. 3:**
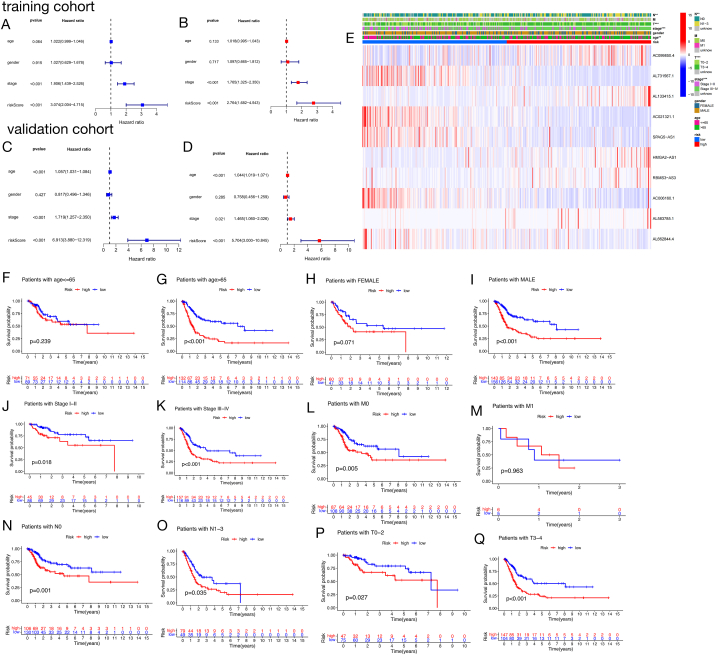
Independent prognostic analysis and subgroup analysis of the FLPS. **(A and B)** Univariate and multivariate Cox regression analyses of the training cohort. (**C and D)** Univariate and multivariate Cox regression analyses of the validation cohort. (**E)** Heatmap of clinical features and FLPS expression levels of the TCGA cohort. (**F–Q)** K-M survival analysis to investigate the applicable BLCA population of the FLPS, including age (cutoff at 65 years of age), gender (female versus male), stage (I versus II), T-stage (T1/2 versus T3/4), N (N0 versus N1/2/3), and M (M0 versus M1).

The prognostic significance of FLPS in BLCA patients was evaluated through univariate and multivariate Cox regression analyses. The training cohort showed a strong correlation between FLPS and OS in the univariate Cox analysis (Fig. 3A), with the multivariate Cox analysis confirming FLPS as a significant prognostic risk factor (Fig. 3B). The results were confirmed in the validation group, confirming the separate and predictive importance of FLPS for individuals with BLCA (Fig. 3C and D).

A stratified analysis of the clinical features was conducted in order to accurately assess the ability of FLPS to predict OS in both subgroups. A heatmap was used to combine the signature-FRLR expression profile and clinical features in TCGA cohort **(**Fig. 3E). The study findings showed that AC099850.4, AL133415.1, HMGA2-AS1, RBMS3-AS1, and AL583785.1 had higher levels of expression in the high-risk group, while AL731567.1, AC021321.1, SPAG5-AS1, AC006160.1, and AL662844.4 had lower levels of expression. The heatmap showed significant differences in clinical features between the high-risk and low-risk groups. Risk scores serve as prognostic indicators regardless of gender, age, tumour size, metastasis status, and lymph node involvement.

Additionally, individuals over the age of 65 with advanced stages of bladder cancer, specifically T and N stages, showed increased risk scores (Supplementary Figs. S7A, C, D, and F). The correlation between risk score and sex and M stage was also significant (Supplementary Figs. S7B and E). Furthermore, survival analysis was performed to explore the applicable subgroups of patients with BLCA of FLPS. The results demonstrated that FLPS was suitable for patients with BLCA aged >65 years but not for patients aged ≤ 65 years (Fig. 3F and G). FLPS was not significant in predicting prognosis in female patients but was meaningful for male patients (Fig. 3H and I). FLPS was substantial in patients with M0 stage disease, but was not significant in patients with M1 stage disease (Fig. 3L and M). We confirmed that the FLPS had a degree of accuracy and reliability in predicting outcomes for patients belonging to various clinical subgroups (Fig. 3J, K, N, O, P, and Q). Therefore, the FLPS risk score was highly correlated with tumour progression and can predict the prognosis of multiple clinical subgroups of patients with BLCA.

### 2.5 Analysis of the biological function and enrichment of the lncRNA prognostic signature associated with ferroptosis

**Fig. 4:**
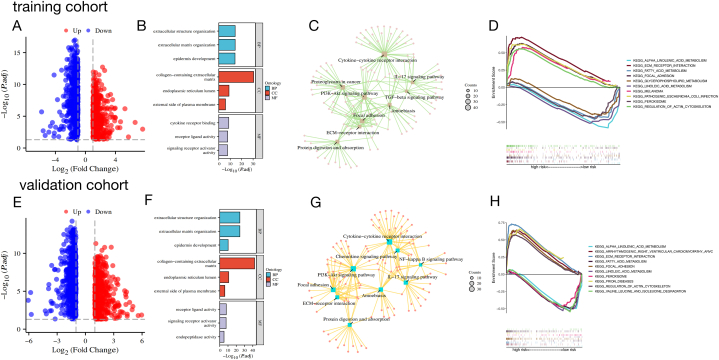
Functional enrichment analysis of differentially expressed gene sets and pathways between high- and low-risk groups. **(A)** Volcano plot of DEGs in the high- and low-risk groups in the training cohort; upregulated and downregulated DEGs are highlighted in red and blue, respectively. (B) GO analysis of the training cohort. (**C)** Visualizations of KEGG analysis in the training cohort. (**D)** The top five most significant pathways using GSEA in the high- and low-risk groups respectively of the training cohort. (**E)** Volcano plot of DEGs in the validation cohort; upregulated and downregulated DEGs are highlighted in red and blue, respectively. (**F)** GO analysis of the validation cohort**. (G)** Visualizations of KEGG analysis in the validation cohort. (**H)** The top five most significant pathways using GSEA in the high- and low-risk groups respectively of the validation cohort.

Due to the varying outcomes seen in patients categorized as high or low risk, we performed an analysis to determine the biological functions, pathways, and gene sets linked to the risk score. The distribution of identified DEGs in the training and validation cohorts is displayed in Fig. 4A and E, which are volcano maps. Both the training and validation cohorts showed significant enrichment of DEGs in gene sets related to tumour-stromal interaction and tumour cell movement and invasion, such as organization of extracellular structures, extracellular matrix (ECM) organization, skin development, and collagen-containing ECM (Fig. 4B and F). The visualisation results of the GO enrichment analysis (Supplementary Figs. S8A and C) confirmed the correlation between the aforementioned gene sets. Moreover, the findings from the analysis of Kyoto Encyclopedia of Genes and Genomes (KEGG) pathways indicated that DEGs were predominantly enriched in pathways related to cytokine−cytokine receptor interaction, ECM receptor interaction, and the PI3K−Akt signalling pathway. The interaction network of enriched pathways is illustrated in Fig. 4C and G (see Supplementary Figs. S8B and D).Additionally, an analysis using Gene Set Enrichment Analysis (GSEA) was performed to explore the biological processes and functions that were enriched in the high-risk score group. In both the training and validation cohorts, pathways related to the tumour microenvironment were found to be more abundant in the high-risk group, such as control of the actin cytoskeleton, interaction with ECM receptors, and focal adhesion. The enhanced pathways in the high-risk category suggest that individuals with BLCA in this group exhibited a greater capacity for tumour proliferation, movement, and infiltration. Various pathways related to metabolism, such as alpha-linolenic acid, fatty acid, and linolenic acid metabolism, showed enrichment in the low-risk group, indicating an increase in protective factors in this group (Fig. 4D and H). The key FRLR of this prognostic signature can regulate cancer progression and outcome by affecting the TME, including the aforementioned biological functions, pathways, and gene sets.

### 2.6 Correlation between the ferroptosis-related lncRNA prognostic signature and the TME

**Fig. 5:**
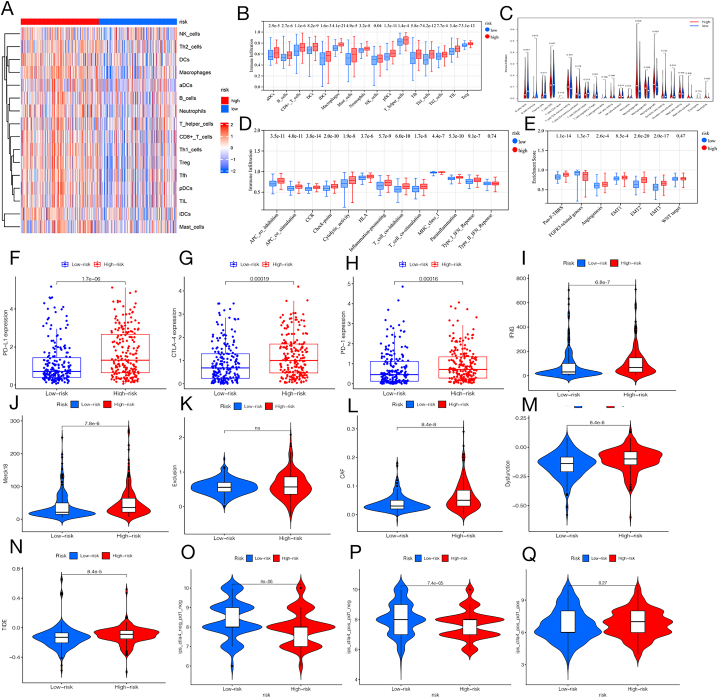
Comparison of TME, immune cells, and immunotherapy between high- and low-risk groups in the TCGA cohort **(A)** Heatmap of immune cells, red indicates high expression, and blue indicates low expression. **(B)** Scores of 16 immune cells using ssGSEA. **(C)** Scores of 22 immune cells using the CIBERSOFT method. **(D)** Different expression levels of immune function-related pathways between the high- and low-risk groups. **(E)** Different expression levels of stroma-related pathways between the high- and low-risk groups. **(F–H)** Different expression levels of immune checkpoint genes (PD-L1, CTLA-4, and PD-1) between the high- and low-risk groups. **(I–N)** Multiple TIDE-related scores of the high- and low-risk groups (IFNG score, Merck18 score, exclusion score, CAF score, dysfunction score, and TIDE score). **(O**–**Q)** Multiple immunotherapy scores of the high- and low-risk groups.

Various examinations delved deeper into the correlation between risk assessment and TME. Initially, we assessed the variations in immune cell infiltration status between the high-risk and low-risk groups. In Fig. 5A, the immune cell fractions distribution was displayed for patients in both high- and low-risk categories. The study found that various immune cells, including activated dendritic cells (aDCs), B cells, CD8^+^ T cells, dendritic cells (DCs), inhibited dendritic cells (iDCs), macrophages, mast cells, neutrophils, NK cells, plasmacytoid dendritic cells (pDCs), T helper cells, follicular T helper cells (Tfh), type 1 T helper (Th1) cells, type 2 T helper (Th2) cells, tumour-infiltrating lymphocytes (TIL), and regulatory T cells (Treg cells), were significantly present in the high-risk group. This suggested that a variety of immune cells were activated in the high-risk group using the single-sample gene set enrichment analysis (ssGSEA) method (Fig. 5B). The CIBERSOFT technique showed that in the low-risk group, there was a higher infiltration of native B cells, plasma cells, follicular T helper cells, regulatory T cells, and activated dendritic cells, while in the high-risk group, M0 type tumour-associated macrophages (TAM), TAM M1, TAM M2, and neutrophils were notably activated. In addition, the low-risk group exhibited a significant presence of anti-cancer immune cells, while the high-risk group showed a substantial presence of various immunosuppressive cells. This could explain the varying outcomes seen in the high-risk and low-risk groups (Fig. 5C). In Fig. 5D, the connection between immune function pathways and FLPS was shown, revealing that APC co-inhibition, APC co-stimulation, CCR, checkpoint, cytolytic activity, HLA, inflammation promotion, MHC class I, para-inflammation, T cell co-inhibition, T cell co-stimulation, and type I IFN response were significantly upregulated in the high-risk group. Fig. 5E shows the correlation analysis between the stromal-related pathway and FLPS, indicating that the pan-fibroblast TGF-beta response signature, angiogenesis, EMT1, EMT2, and EMT3 were decreased in the low-risk group, while FGFR3-related genes were increased in the low-risk group. Additionally, we assessed the levels of TGF-beta and genes related to the epithelial-mesenchymal transition (EMT) pathway, as well as immune activation genes, in order to illustrate the connection between FLPS and TME-related pathways in both high- and low-risk groups. In the high-risk group, several genes such as COL4A1, PDGFRA, TGFB2, TWIST1, VIM, and ZEB1 showed increased expression, while SMAD9 exhibited decreased expression (Supplementary Fig. S9A). Likewise, several genes related to immune response (specifically CD8A, CXCL10, GZMA, GZMB, IFNG, PRF1, and TNF) showed increased levels of expression in the high-risk category, except for TBX2 (Supplementary Fig. S9B). Patients classified as high-risk exhibited elevated estimated immune and stromal scores, as shown in Supplementary Figs. S9C and E. These results demonstrated that the ten key FRLR-based signature could regulate immune cell function and inflammation, affect the expression of immune-related genes and signalling pathways, and modulate the immune infiltration status and stromal microenvironment of BLCA, implying that the key FRLRs may serve as critical immunotherapeutic targets in treating BLCA.

### 2.7 The ferroptosis-associated lncRNA prognostic signature's predictive value for immunotherapy efficacy in BLCA

To explore whether FLPS can be used as a predictor of BLCA immunotherapy, we investigated the correlation between FLPS and immunotherapy-related indicators. The high-risk group exhibited elevated expression levels of several immune checkpoint genes (PD-L1, CTLA4, and PD-1), which are crucial targets for immunotherapy. The findings indicated that FLPS may play a role in predicting the effectiveness of immunotherapy (Fig. 5F–H). Additionally, the high-risk group exhibited elevated scores for cancer-associated fibroblast, IFNG, and Merk18, as determined by tumour immune dysfunction and exclusion (TIDE) scores (Fig. 5I, J, and L). The high-risk group exhibited a high dysfunction score, which serves as an indicator of immune dysfunction in cancer (Fig. 5M). Likewise, the TIDE score, which signifies immune evasion, was elevated in the group at high risk (Fig. 5N). However, the exclusion score, which is an indicator of immune exclusion in cancer, was unrelated to the risk score (Fig. 5K). The findings indicated increased immunosuppressive markers in the high-risk group, along with elevated immune dysfunction and immune evasion in patients classified as high-risk. The IPS, a measure of how well immunotherapy works for patients in the TCGA group, was utilized to assess the effectiveness of FLPS in predicting immunotherapy outcomes for BLCA patients. In the low-risk group, the combined IPS and IPS for CTLA-4 inhibitors were notably greater compared to the high-risk group, indicating that BLCA patients with lower risk scores are likely to have improved responses to immunotherapy, particularly with CTLA-4 inhibitors (Fig. 5O and P). However, the IPS for PD1 blockers did not differ significantly between the two groups (Fig. 5Q). These results further verify the significance of FLPS in predicting the effectiveness of immunotherapy in patients with BLCA.

### 2.8 Identification of the vital signature-FRLR and further investigation of the biological pathways and mechanisms regulated by HMGA2-AS1

We investigated the importance of FLPS in predicting the outcome and response to immunotherapy in BLCA patients, then explored the potential biological pathways and mechanisms of ferroptosis through the lens of the key signature FRLR. Initially, we screened key signature-FRLRs with congruent expression and prognostic conditions that were upregulated in tumour tissues and as risk factors for the opposite condition. HMGA2-AS1, AC099850.4, and AL662844.4 were filtered and processed for selection. We correlated gene expression with clinical traits, and selected genes with higher expression levels that were associated with poorer clinical traits (stage and grade). The results indicated that HMGA2-AS1 is a crucial signature FRLR that is highly expressed in patients with BLCA with poorer clinical traits (Supplementary Fig. S10). In our comprehensive analysis combined with the expression of FRLR s and clinical prognosis results, the high expression of HMGA2-AS1 predicted a worse clinical prognosis, suggesting that HMGA2-AS1 has a greater potential as a diagnostic marker and a therapeutic target, so we chose HMGA2-AS1 as the vital signature-FRLR and performed the subsequent analyses.

**Fig. 6:**
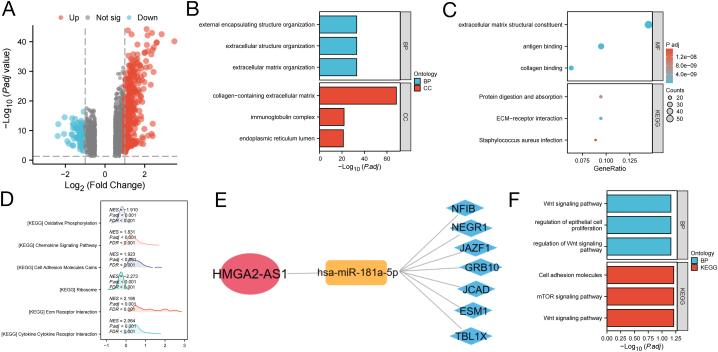
Investigation of biological pathways and mechanisms regulated by HMGA2-AS1. **(A)** Volcano plot of DEGs in the high- and low- HMGA2-AS1 expression groups; the red point indicates the gene with a log2FC > 1 and Log2FC < −1, whereas the blue point indicate and the opposite. **(B–C)** GO and KEGG enrichment analysis of the DEGs. **(D)** The top six most significant pathways using GSEA in the high- and low- HMGA2-AS1 expression groups. **(E)** A key signature-FRLR ceRNA network of one lncRNA (red), one target miRNA (yellow), and seven target mRNAs (blue). **(F)** Enrichment analysis results of GO-BP and KEGG of the seven target mRNAs

DEGs were identified by conducting differential expression analysis between groups with high and low expression levels of HMGA2-AS1. Fig. 6A (volcano map) shows the distribution of the DEGs. Following this, GO and KEGG examinations were performed to clarify the biological pathways linked to DEGs. The findings showed that the DEGs were notably concentrated in pathways related to ECM, such as ECM arrangement, ECM with collagen, components of ECM structure, and interaction with ECM receptors. These findings indicate that HMGA2-AS1 could potentially influence the biological activity of the ECM, as shown in Fig. 6B and C. The results were further validated using subsequent GSEA. The results suggest that the pathway involving EMT receptors is highly upregulated in individuals with high expression of HMGA2-AS1 (Fig. 6D). Enrichment analysis revealed a crucial role of HMGA2-AS1 in EMT regulation. Next, we established a ceRNA network to investigate the regulatory mechanisms of HMGA2-AS1.Within the ceRNA network, we discovered a single miRNA along with seven mRNA nodes (Fig. 6E). Analysis of GO and KEGG functions showed that the seven mRNA targets primarily participated in the Wnt signalling pathway, epithelial cell proliferation regulation, cell adhesion molecules, and the mTOR signalling pathway (Fig. 6F). A prior investigation indicated that the Wnt pathway plays a vital role in controlling EMT, suggesting that HMGA2-AS1 could impact EMT by modulating the Wnt signalling pathway through a ceRNA mechanism [21].

### 2.9 Drug sensitivity and immunotherapy analysis of the ceRNA networ

After elucidating the potential biological pathways and mechanisms of ferroptosis from the perspective of the vital signature-FRLR, we performed drug sensitivity and immunotherapy analyses of the ceRNA network to identify possible new targets for clinical treatment.

**Fig. 7:**
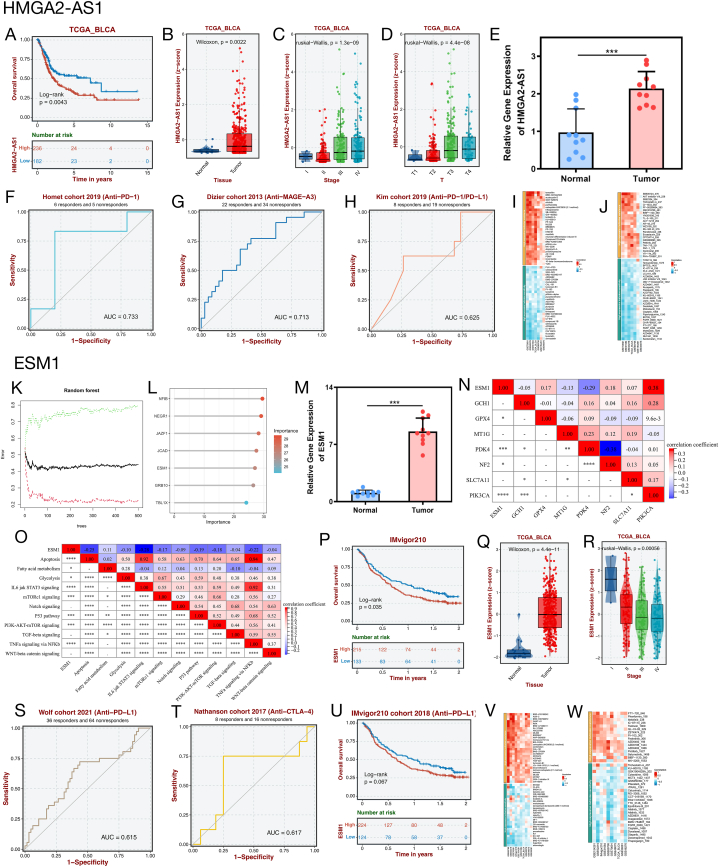
Drug sensitivity and immunotherapy analysis of the ceRNA network **(A)** K-M survival curve to show the OS of patients with BLCA in low HMGA2-AS1 expression and high EMX2OS expression subgroups. **(B)** The expression levels of HMGA2-AS1 in validation set TCGA_BLCA. **(C)** The expression levels of HMGA2-AS1 in validation set TCGA_BLCA among different tumour stages. **(D)** The expression levels of HMGA2-AS1 in validation set TCGA_BLCA among different T stages. **(E)** The expression level of HMGA2-AS1 detected using RT-qPCR in clinical sample. **(F–H)** ROC curves of anti-PD1、anti-PD- L1 and anti-MAGE-A3 based on HMGA2-AS1 expression. **(I**–**J)** Drug sensitivity analysis based on HMGA2-AS1 expression. **(K and L)** Identification top 5 mRNAs (NFIB, NEGR1, JAZF1, JCAD, and ESM1) based on random forest algorithm. **(M)** The expression level of ESM1 detected using RT-qPCR in clinical sample. **(N)** Correlation analysis of ESM1 with hub FRGs. **(O)** Correlation analysis of ESM1 with tumour-related pathways. **(P)** K-M survival curve to show the OS of patients with BLCA in low ESM1 expression and high ESM1 expression subgroups. **(Q)** The expression levels of ESM1 in validation set TCGA_BLCA. **(R)** The expression levels of ESM1 in validation set TCGA_BLCA among different tumour stages. **(S**–**T)** ROC curves of anti-PD- L1 and anti-CTLA-4 based on ESM1 expression. **(U)** K-M survival curve to show the OS of anti-PD-L1 based on ESM1 expression. **(V–W)** Drug sensitivity analysis based on ESM1 expression. *p < 0.05, **p < 0.01, ***p < 0.001, and ****p < 0.0001.

Within the TCGA cohort, analysis of the Kaplan-Meier survival curve revealed that individuals with elevated levels of HMGA2-AS1 had a poorer overall survival compared to those with lower levels (Fig. 7A, p = 0.0043). Furthermore, HMGA2-AS1 exhibited high expression in tumour tissues, suggesting notable variations in expression levels among various stages and T stages (Fig. 7B–D). RT-qPCR analysis of clinical samples showed increased HMGA2-AS1 expression in tumour tissues compared to normal tissues, indicating a significant difference, while miRNAs followed a similar pattern (Fig. 7E, p < 0.001). Furthermore, based on the ROC curves (Fig. 7F–H), the AUC values were 0.733, 0.713, and 0.625 for anti-PD-1 (Homet cohort 2019), anti-MAGE-A3 (Dizier cohort 2013), and anti-PD-1/PD-L1 (Kim cohort 2019) antibodies, respectively. HMGA2-AS1 expression effectively predicts the efficacy of immunotherapy. Based on drug sensitivity-related databases (GDSC1, GDSC2, CTRP1, and PRISM), the results demonstrated that highly expressed HMGA2-AS1 was sensitive to dasatinib and pazopanib (Fig. 7I and J), which are clinically used for cancer treatment, suggesting that the application of dasatinib and pazopanib in BLCA treatment may potentially affect BLCA progression by inhibiting HMGA2-AS1 expression. The importance of functional mRNAs downstream of hsa-miR-181a-5p was identified using the random forest algorithm of machine learning, and the top five important ones were NFIB, NEGR1, JAZF1, JCAD, and ESM1 (Fig. 7K and L), and the Gene expression was correlated with survival prognosis, and the genes with higher expression were selected to be associated with worse survival prognosis. Within the IMvigor210 cohort, it was observed that the ESM1 high-expression group had a poorer overall survival compared to the low-expression group, as indicated by the Kaplan-Meier survival curves (Fig. 7P, p = 0.035). Conversely, in the TCGA cohort, tumour tissues exhibited high levels of ESM1 expression, with notable variations in expression levels across different stages (Fig. 7Q and R). The RT-qPCR findings indicated a substantial increase in ESM1 expression in tumour tissues compared to normal tissues, showing a nearly tenfold difference in expression levels between the two types of tissue (Fig. 7M, p < 0.001). Based on these results, ESM1 was identified as a crucial mRNA and we subsequently explored its biological functions of ESM1.DEGs were identified between high- and low-expression groups of ESM1 using differential expression analysis. Supplementary Fig. S11A displays the DEGs distribution in a volcano plot. The biological pathways related to DEGs were investigated through GO and KEGG analyses. The findings indicated that the differentially expressed genes (DEGs) were notably concentrated in pathways related to the immune system, indicating a potential role of ESM1 in immune function (Supplementary Figs. S11B and C). The GSEA results revealed that ESM1 was associated with TME-related pathways (Supplementary Fig. S11D). We conducted correlation analysis to delve deeper into the biological roles of ESM1. Fig. 7N shows the correlation analysis of ESM1 with the central FRGs, demonstrating that ESM1 correlated with central FRGs, including GPX4, PDK4, NF2, and PIK3CA, among which ESM1 exhibited a strong positive correlation with PIK3CA. Fig. 7O shows the correlation analysis of ESM1 with tumour-related pathways, revealing that ESM1 was closely associated with several tumour-related pathways, ESM1 had a strong negative correlation with IL6 jak STAT3 signalling, and ESM1 had positive correlated with fatty acid metabolism. The findings suggested a strong connection between ESM1 and ferroptosis, impacting tumour advancement and potentially influencing the development of BLCA. This could offer innovative approaches for BLCA treatment by targeting the ferroptosis pathway. In order to investigate the potential benefits of ESM1 in medical care, assessments were conducted on drug responsiveness and immunotherapy. Based on the ROC curves (Fig. 7S and T) (Fig. 7S and T), the AUC values for anti-PD-L1 (Wolf cohort 2021) and anti-CTLA-4 (Nathanson cohort 2017) were 0.615 and 0.617, respectively. ESM1 expression was a strong predictor of immunotherapy response in the anti-PD-L1 group from the IMvigor210 cohort in 2018. Patients with high ESM1 levels had a poorer overall survival compared to those with low levels (p = 0.067, Fig. 7U). Based on pharmacovigilance-related databases (GDSC1, GDSC2, CTRP1, and PRISM), high ESM1 expression was sensitive to erismodegib and olaparib (Fig. 7V and W), which have antitumour potential, suggesting that erismodegib and olaparib may also be applicable in BLCA treatment to influence tumour development by inhibiting ESM1 expression. For the four other important mRNAs, NEGR1, JAZF1, JCAD, and NFIB, we also performed K-M survival, expression level, immunotherapy efficacy, and drug sensitivity analyses (Supplementary Fig. S12). In conclusion, the establishment of a ceRNA network has significant implications for the prediction of the prognosis of BLCA patients and the provision of innovative therapeutic approaches.

## 3 Discussion

Ferroptosis has been linked to the creation of beneficial immune reactions in different types of cancer [22,23]. Cancer cells resist ferroptosis by downregulating tumour suppressor molecules (p53, BAP1, and KEAP1), which hinders the production and oxidation of PUFA-PL (ACSL4 and POR), and triggering defense mechanism to promote tumour progression and metastasis [20]. Accordingly, methods that regulate ferroptosis in tumour cells are becoming novel therapies for cancer treatment. With the rapid development of functional genomic techniques, the significant role of lncRNAs in cancer has attracted wide attention [24]. lncRNAs can affect tumour ferroptosis by regulating lipid metabolism, ROS accumulation, and iron-dependent oxidative stress [25,26]. Wang et al. confirmed that lncRNA LINC00336, a sponge for the endogenous miRNA MIR6852, downregulates CBS expression to promote ferroptosis in lung cancer [27]. Yu et al. reported that lncRNA XAV939 downregulates SLC7A11 to increase iron concentration and inhibit NSCLC development by activating the ferroptosis-mediated pathway [28]. Given the involvement of lncRNAs in ferroptosis regulation, it is essential to thoroughly examine the prognostic value of FRLR in managing BLCA.

The present study constructed ten FRLRs (AC099850.4, AL731567.1, AL133415.1, AC021321.1, SPAG5-AS1, HMGA2-AS1, RBMS3-AS3, AC006160.1, AL583785.1, and AL662844.4). Among these ten FRLRs, HMGA2-AS1 and AC099850.4, were upregulated and prognostic risk factors, whereas AL662844.4 a prognostic protective factor, was downregulated in BLCA. However, the expression trends and prognostic contributions of other FRLRs (AL731567.1, AL133415.1, AC021321.1, SPAG5-AS1, RBMS3-AS3, AC006160.1, and AL583785.1) in BLCA were inconsistent. Alterations in the expression levels of HMGA2-AS1, AC099850.4, AL662844.4, AL133415.1, and RBMS3-AS3 were associated with clinical prognosis in other tumours, suggesting the potential value of lncRNAs in predicting tumour prognosis. Ros et al. reported that the HMGA2-AS1 transcript aggravates the malignancy of pancreatic cancer by positively regulating HMGA2 expression and upregulating the migration characteristics of tumour cells [29]. The lncRNA AC099850.4 showed increased expression levels in various tumours and was associated with a negative prognosis [30,31]. The lncRNA AL662844.4, an enhancer-related lncRNA, enhances immune function by upregulating H3K27AC expression in cancers [32]. The AL133415.1 gene has been linked to negative clinical results due to its role in increasing resistance to immunotherapy, as reported in a study [33]. RBMS3-AS3 overexpression inhibited tumour cell proliferation, migration, invasion, angiogenesis, and tumourigenicity [34]. The functions of AL731567.1, AC021321.1, SPAG5-AS1, AC006160.1, and AL583785.1 in BLCA require further study.

Recently, there has been a growing interest in the biological functions and molecular biological processes that signature-FRLRs influence, in addition to their predictive ability. Therefore, we conducted the following experiments: Functional enrichment analysis revealed that DEGs in the high-risk group were enriched in TME-related pathways, including cytokine-cytokine receptor interactions, actin cytoskeleton regulation, ECM receptor interactions, epidermal development and collagen-containing ECM. Studies have suggested that dysregulation of these pathways is associated with cancer cell proliferation, tumour metastasis, and immunosuppression [[35], [36], [37]] and leads to poor cancer prognosis, which is consistent with our findings. Furthermore, the ECM modulator ITIH5 affects cell adhesion and motility in BLCA [38]. Wu et al. found that the dysregulation of EMT-related genes contributes to drug resistance in BLCA. The results indicate that FLPS plays a role in controlling pathways related to the tumor microenvironment in BLCA [39]. To explore the specific functions of FLPS in the TME, we analysed the correlations between FLPS and several characteristic pathways or gene sets in the TME. ssGSEA findings showed that the high-risk group had notably elevated expression levels of different immune cells and pathways related to immune function compared to the low-risk group. CIBERSOFT results revealed that immunosuppressive immune cells (TAM M0, TAM M2, activated dendritic cells, and neutrophils) were upregulated in the high-risk group [40,41]. Recent research has shown that a large number of TAMs and tumor-associated neutrophils are closely linked to negative outcome in BLCA patients and play a role in cancer evading the immune system [42,43], indicating that the high infiltration of immunosuppressive immune cells in the high-risk group may contribute to poor prognosis. Tumour proliferation-related pathways, including angiogenesis, the EMT1 pathway, and the EMT3 pathway, were upregulated in the high-risk group. This result suggests that blood vessel and matrix-generating activities were significantly upregulated in the high-risk group, contributing to tumour progression and causing poor clinical outcomes [44]. Moreover, well-known immune checkpoint genes such as PD-1, CTLA-4, and PD-L1 are critical therapeutic targets for immunotherapy [[45], [46], [47]]. Elevated expression levels of all three genes were found in the high-risk group in this study. Our results indicated that signature-FRLRs were involved in regulating TME components, and investigating immune cell- and stromal-related pathway distribution in individuals provided fundamental insights into tumour prognosis and immunotherapy in BLCA.

To further explore the predictive value of FLPS for BLCA immunotherapy, we analysed FLPS in combination with immunotherapy-related data (TIDE-related scores and TCGA-IPS). High levels of.

CAF and Merk18 expression, signaling poor response to immunotherapy, were significantly increased in the high-risk cohort. Patients in the high-risk groups showed increased exclusion and TIDE scores, suggesting that those with high-risk scores had compromised immune function. Furthermore, our analysis revealed that individuals classified as high-risk exhibited a reduced IPS, validating the ability of FLPS to anticipate the response to immunotherapy in BLCA patients. In summary, our results provide guidelines for the development of BLCA immunotherapy.

HMGA2-AS1 is a vital signature-FRLR. The GO and KEGG enrichment analyses and GSEA revealed the potential role of HMGA2-AS1 in EMT. The ceRNA network, including HMGA2-AS1 (lncRNA), has-miR-181a-5p (miRNA), NFIB, NEGR1, JAZF1, GRB10, JCAD, ESM1, and TBL1X (mRNAs), was obtained to study the role of HMGA2-AS1 in BLCA. The functions of the seven target mRNAs in the Wnt pathway were demonstrated using enrichment analysis. Thus, combined with the ceRNA network, the role of HMGA2-AS1 in EMT was verified, indicating that HMGA2-AS1 may influence EMT by regulating the Wnt signalling pathway via the ceRNA mechanism. These results reveal the crucial role of HMGA2-AS1 in EMT; however, no studies have focused on the role of HMGA2-AS1 in EMT. However, other genes in the ceRNA network were found to be involved in EMT-related pathways. Has-miR-181a-5p drove EMT in advanced retinoblastoma to enhance its invasion and migration [48]. ESM1 accelerates cervical cancer cell proliferation and migration by promoting EMT progression [49]. Rivero et al. indicated that TBL1 is required for the mesenchymal phenotype of transformed BLCA cells [50]. Therefore, HMGA2-AS1 can regulate EMT in BLCA, and this ceRNA network may be possibly one of a regulatory mechanism. The study offers fresh perspectives on the possible molecular and regulatory pathways of HMGA2-AS1, directing future research endeavors.

We screened crucial mRNA for subsequent analysis. Endothelial cell-specific molecule 1 (ESM1), also called endocan, is a proteoglycan that is secreted by endothelial cells and has a weight of 50 kDa [51]. ESM1 is overexpressed in various tumours, including lung cancer, esophageal cancer, hepatocellular carcinoma, and ovarian cancer [[52], [53], [54], [55]]. Elevated levels of ESM1 were detected in the serum and urine of BLCA patients [56], and aberrant ESM1 expression was also observed in BLCA, which is consistent with our findings. ESM1 possesses angiogenic and inflammatory characteristics that could impact vascular permeability and is crucial in the advancement of tumours through the control of vascular pathways. ESM1 is crucial for regulating several molecular signalling pathways that are directly involved in tumour development and progression. The signalling pathways involved in ESM1 include AKT/NF-kappaB/Cyclin D1, Wnt/β-catenin, DLL4-Notch, AKT/eNOS, and NFkB/iNOS, and other important pathways [[57], [58], [59], [60]]. Our findings align with prior research indicating a strong connection between ESM1 and various pathways related to tumours, particularly with a notable link to IL6/jak/STAT3 signaling. IL6/jak/STAT3 signalling is crucial in the development and advancement of tumours, and elevated IL6 over-activates the jak/STAT3 signalling pathway, which often causes poor clinical prognosis [61,62]. In the TME, this signalling pathway promotes tumour cell proliferation, invasion, and metastasis while strongly suppressing the antitumour immune response and is thus considered an important target for tumour therapy [63]. ESM1 is closely related to the IL6/jak/STAT3 signalling pathway, and enrichment analysis demonstrated that ESM1 is associated with several TME-related pathways, such as cytokine–cytokine receptor interactions and cell adhesion molecules. Furthermore, our results suggest that ESM1 is closely related to hub FRGs, and ESM1 may be involved in ferroptosis. Consequently, ESM1 could potentially impact the development and progression of tumours through a sophisticated and tightly controlled process, making it a hopeful target for treating BLCA alongside immunotherapy and ferroptosis. Immunotherapy has become a disruptive cancer treatment strategy, and the search for reliable biomarkers to predict patient response to immunotherapy is one of the hotspots of current research. ESM1 and HMGA2-AS1 may serve as important markers for predicting the efficacy of immunotherapy in patients with BLCA. Drug sensitivity analysis of ESM1 and HMGA2-AS1 revealed that HMGA2-AS1 was sensitive to dasatinib and pazopanib, whereas ESM1 was sensitive to erismodegib and olaparib. Dasatinib is a targeted therapy used primarily for the treatment of chronic myeloid leukaemia (CML) and Philadelphia chromosome-positive acute lymphoblastic leukaemia (ALL) [64,65]. Pazopanib inhibits angiogenesis in tumours and is used to treat advanced/metastatic renal cell carcinomas and advanced soft-tissue sarcoma [66,67]. Erismodegib inhibits tumour cell growth and is used to treat basal cell carcinoma [68]. Olaparib may potentiate the cytotoxicity of DNA-damaging agents and reverse chemoresistance and radioresistance in tumour cells for the treatment of advanced ovarian cancer [69]. These agents may have potential applications in the treatment of BLCA, based on their different mechanisms of action and known clinical applications. For instance, the targeted effect of dasatinib may be efficacious in certain instances of BLCA, whereas the anti-angiogenic properties of pazopanib may be instrumental in the treatment of BLCA. Erismodegib and Olaparib, on the other hand, may affect the growth and drug resistance of BLCA cells via different pathways. The findings serve as a foundation for the advancement of personalized healthcare and targeted treatment. Nevertheless, further clinical trials are required to assess the efficacy and safety of these drugs for the treatment of BLCA. Such studies will further guide treatment strategies for patients with BLCA and improve treatment outcomes.

As a matter of fact, there have been some studies screening ferroptosis -related lncRNAs associated with the prognosis of BLCA [70,71], compared with these studies, we have enhanced our comprehension of the molecular mechanisms of these lncRNAs in BLCA by constructing a ceRNA network. In addition, we performed immunotherapy analysis and drug sensitivity analysis based on the ceRNA network, confirmed its prediction of immunotherapy efficacy for BLCA, and screened first-line anticancer drugs that have been applied in the clinic and are expected to improve the prognosis of BLCA.

## 4 Conclusions

In conclusion, we demonstrated that FRGs play a crucial role in BLCA, developed the FLPS with predictive power independent of other common clinical factors, and demonstrated its validity for predicting OS in patients with BLCA. Furthermore, we investigated the role of signature-FRLRs, which indicated their essential role in regulating cancer progression and prognosis by affecting the TME, including the aforementioned biological functions, pathways, and gene sets. Additionally, FLPS has been found to efficiently predict immunotherapy in BLCA, which has significant implications for clinical treatments. Finally, we constructed a ceRNA regulatory mechanism based on key signature FRLRs and performed immunotherapy and drug sensitivity analyses. Our findings suggest that patients with BLCA are more susceptible to four drugs (dasatinib, pazopanib, erismodegib, and olaparib). Future studies should validate the gene signature and predictive accuracy of the BLCA immunotherapy target based on FRLRs in additional external and immunotherapy cohorts.

## 5 Limitations and perspectives

This study had some limitations. First, our study had limitations in terms of sample sources, potentially affecting external validity. The absence of sex and ethnicity information in the sample could compromise the generalisability of our findings. To address this issue, future studies should consider more diverse and representative samples.

Second, further functional experiments are crucial to validate the conclusions of this study. These experiments may involve in vitro cellular studies or animal model investigations to ascertain whether FRLRs directly influences key biological processes, such as tumour cell proliferation, migration, and invasion. The precise role of these RNAs in cancer biology can be elucidated through gene silencing or overexpression experiments.

Moreover, the application of the four drugs identified in our BLCA screening process for BLCA could be explored in more detail. This involves adding these drugs to the cancer cell culture medium and evaluating their impact on various aspects of cancer cells, including proliferation, apoptosis, migration, and invasion. This additional experiment will provide a more comprehensive understanding of the potential therapeutic applications of these drugs in BLCA.

## 6 Materials and methods

### 6.1 Data collection

The RNA-seq transcriptome data and mature miRNA sequencing data of 414 BLCA tissues and 19 adjacent normal tissues and the corresponding clinical and prognostic data were downloaded from the TCGA database (https://portal.gdc.cancer.gov/). After excluding the tissues without clinical information, 412 BLCA tissues were enrolled for further study, and the overall clinical details are shown in Supplementary Table S1. The RNA-seq transcriptome GSE39281, which contains 94 BLCA samples with prognostic information, was extracted from the (GenExpression Omnibus GEO (; https://www.ncbi.nlm.nih.gov/) database. The scRNA dataset GSE135337, which contains single-cell mRNA profiles of seven primary tumour samples and one paracancerous tissue sample from seven patients with BLCA, was downloaded from the GEO database. In addition, a local cohort that included 65 samples was obtained from the Urology Bladder Tumour Database of the First Affiliated Hospital of Wenzhou Medical University. BLCA samples from the local cohort were collected from 2022 to 2023, with OS as the main measure of survival time.

The efficacy of the genes in the immunotherapy cohort was predicted using the BEST database (https://rookieutopia.com/app_direct/BEST/). Nine immunotherapy cohorts were included in the study. The Homet cohort included six responders and five non-responders; the Dizier cohort included 22 responders and 34 non-responders; the Kim cohort included eight responders and 19 non-responders; the Ascierto cohort included four responders and seven non-responders; the Gao cohort included three responders and 34 non-responders; the Nathanson cohort included eight responders and 16 non-responders; the Wolf cohort included 36 responders and 64 non-responders; the Hugo cohort included 27 samples with prognostic information; and the Imvigor210 cohort included 348 samples with prognostic information.

259 FRGs were obtained from previous studies [72,73], presented in Supplementary Table S2. The TIDE algorithm (http://tide.dfci.harvard.edu) was utilized to calculat immunotherapy-associated scores for individuals within the TCGA dataset. In addition, IPS was downloaded from the TCGA database.

### 6.2 Processing and analysis of scRNA-seq data and protein-protein interaction analysis

Seven scRNA-seq data of primary bladder tumour samples were included in the single-cell data analysis, and the “seurat” R package (version 4.2.3) was implemented to construct the single-cell data processing object. To avoid errors caused by ineligible cells, cells were filtered with a gene expression number per cell between 500 and 6000. Highly variable genes were identified in each cell after normalising the scRNA-seq data and controlling for the relationship between average expression and variance. PCA was performed on scRNA-seq data, selecting the top 20 principal components (PCs) for subsequent analyses. The UMAP algorithm conducts a comprehensive analysis of dimensionality reduction on the top 20 principal component pairs of the samples. Using the "singleR" package and combining the previous research to annotate different clusters [74]. The DEGs for a given cell type were determined using the FindAllMarkers function, and a violin plot was used to visualise the top five DEGs of each cell population. The exploration of interactive relationships among 259 FRGs and identification of eight hub FRGs was conducted through protein-protein interaction analysis [75]. Finally, dot plots were constructed to display the expression levels of the eight hub FRGs in each cell population.

### 6.3 Establishment and validation of the ferroptosis-related lncRNA prognostic signature

The "limma" R package was used to calculate the correlation coefficient between FRGs and lncRNAs in the TCGA cohort, and lncRNAs with correlation coefficient |R| > 0.6 and FDR <0.001 were screened as FRLRs. We then performed univariate Cox analysis to screen prognostic FRLRs with FDR <0.01, based on the expression levels of the identified FRLRs and the OS of patients with BLCA in TCGA cohort. Subsequently, all samples were randomly divided into training (n = 204) and validation (n = 202) cohorts. LASSO regression was then performed via R software package “glmnet”. After 1000 iterations of LASSO regression, the best lambda and most critical prognostic FRLRs were identified, and the FLPS was constructed in the training cohort. The risk score can be calculated using the following formula: RiskScore = e ^∑Coef(i) X(i); Coef(i) is the coefficient, χ(i) is the FPKM value of each signature-FRLR.

Subsequently, patients were stratified into high- and low-risk groups according to the median risk score. The Kaplan-Meier survival curve was employed to assess the prognosis via the R packages "survival" and "survminer.". Time-dependent ROC curves were plotted using the “timeROC” R package to assess the predictive power of the signature. PCA was performed using the “stats” R package. T-SNE, produced using the “Rtsne” R package, explored group distribution.

Functional enrichment analysis of differentially expressed gene sets and pathways between high- and low-risk groups.

The R package “limma” was used to identify differential DEGs between high- and low-risk groups, with |log2FC| > 1 and FDR <0.05 as the criterion. DEGs were subjected to GO and KEGG enrichment analyses to explore the potential biological functions of FLPS. GSEA, performed using GSEA 4.1.0 (http://www.broad.mit.edu/gsea/), was used to compare the biological pathways that were significantly different between the high- and low-risk groups [76].

### 6.4 Acquisition of infiltration scores of TME related pathways and cells

Infiltration scores of 16 types of immune cells, 13 types of immune-related pathways, and seven types of stromal-related pathways were calculated using ssGSEA using the “gsva” R package. The gene sets of the 16 immune cells, 13 immune-related pathways, and seven stromal-related pathways are presented in Supplementary Table S3. Another method, CIBERSOFT, was utilized to calculate infiltration scores of 22 types of immune cells via the “CIBERSOFT” R package. Additionally, three TME-related scores (immune score, stromal score, and estimate score) were calculated via the “ESTIMATE” R package.

### 6.5 Single gene bioinformatic analysis and construction of the ceRNA network

Single-gene bioinformatics analyses included differential expression analysis, GO and KEGG enrichment analysis, and GSEA. Based on the median expression value of the key signature FRLR, patients with BLCA were divided into high- and low-expression groups. Genes with |log2FC| > 1 and FDR <0.05 were identified as DEGs between the high- and low-expression groups using the R package “limma”, and these DEGs were then enrolled in GO and GSEA enrichment analysis. GSEA was used to compare the biological pathways that were significantly different between the high- and low-expression groups.

Construction of ceRNA network. First, DIANA tools (https://diana.e-ce.uth.gr/lncbasev3/interactions/) were used to predict lncRNA-miRNA pairs. Differential expression analysis was then implemented to obtain differentially expressed miRNAs (|log2FC| > 1 and FDR <0.05) in the TCGA cohort using R package “limma”. Next, we identified the intersection between predicted and differentially expressed miRNAs. Subsequently, the predicted miRNA-mRNA pairs were retrieved from the miRWalk database (http://mirwalk.umm.uni-heidelberg.de/). The prediction criteria were TargetScan, miRDB, and a score of 1. Differentially expressed mRNAs were identified with |log2FC| > 1 and FDR <0.05 in the TCGA cohort using R package “limma”. Similarly, we identified the intersection of the predicted and differentially expressed mRNAs. Finally, we established a ceRNA network by matching the lncRNA-miRNA and miRNA–mRNA pairs. The random forest algorithm in machine learning was used to evaluate the importance the genes that affect the prognosis of patients with BLCA based on “randomForest” R package.

### 6.6 RT-qPCR analysis

To verify the reliability of the lncRNA prognostic score in patients with BLCA, TRIzol reagent (Invitrogen, USA) was used to extract total RNA from tumour tissue and normal control tissue, and cDNA samples were synthesised using a Superscript II first-strand cDNA synthesis kit (TaKaRa, Japan). The expression levels of HMGA2-AS1 and ESM1 were quantified using real-time fluorescence. GAPDH is used as an internal control.

**Table:** 

	Forward:5′ to 3′	Reverse:3′ to 5′
HMGA2-AS1	ACTTGGTAGCACACAGGAGG	CATCTGCCTTCCCTCCAACA
ESM1	TTGCTACCGCACAGTCTCAG	GCCATGTCATGCTCTTTGCAG
GAPDH	AATGGGCAGCCGTTAGGAAA	GCGCCCAATACGACCAAATC

### 6.7 Statistical analysis

All statistical analyses were performed using R software (version 4.1.0) and the accompanying software packages. The RNA-seq data (FPKM values) were normalised to log2 (FPKM+1) for differential expression analysis. The Wilcoxon test was used to analyse the differentially expressed FRLRs between cancer and adjacent normal tissues. Spearman's correlation coefficient test was used to assess the correlations among different variables. Univariate and multivariate COX regression analyses were performed to identify the independent risk factors. Kaplan-Meier survival curves were constructed to show the survival differences between the different groups. The performance of the signature was assessed using time-dependent ROC analysis and AUC. The Mann-Whitney *U* test was used to compare differences in ssGSEA scores of immune cells or pathways between the high- and low-risk groups. Statistical significance was set at P < 0.05.

## Funding

This work was supported by the 10.13039/501100002858China Postdoctoral Science Foundation (2023M743009), 10.13039/501100004835Zhejiang University of Stomatology Postdoctoral Scientific Research Foundation (2023PDF013), 10.13039/501100004731Zhejiang Provincial Natural Science Foundation of China under Grant (LY21H050006) and National College Students' innovation and entrepreneurship training program (202210343028).

## Ethics approval and consent to participate

The Human Research Ethics Committee of the First Affiliated Hospital of Wenzhou Medical University reviewed and approved the research involving human participants. The participants gave their written informed consent to take part in this study. The ethics number was KY2022-069 (approval date: 2022- 05–19).

## Consent for publication

Not applicable.

## Data availability statement

The datasets generated and/or analysed during the current study are available in the TCGA database (https://portal.gdc.cancer.gov/) (TCGA-BLCA) and GEO database (https://www.ncbi.nlm.nih.gov/) (GSE135337). And the data that support the findings of this study are available from the corresponding author upon reasonable request.

## CRediT authorship contribution statement

**Zhan Yang:** Writing – review & editing, Writing – original draft, Visualization, Validation, Software, Resources, Methodology, Investigation, Formal analysis, Data curation, Conceptualization. **Xiaoqi Li:** Visualization, Validation, Software, Resources, Methodology, Conceptualization. **Lijun Zhou:** Writing – original draft, Validation, Resources, Methodology, Formal analysis, Data curation. **Yaxian Luo:** Writing – review & editing, Writing – original draft, Validation, Software, Resources, Formal analysis, Data curation. **Ning Zhan:** Software, Investigation, Data curation. **Yifan Ye:** Software, Resources, Methodology. **Zhichao Liu:** Validation, Project administration, Conceptualization. **Xiaoting Zhang:** Visualization, Software, Formal analysis. **Tao Qiu:** Software, Resources, Formal analysis. **Lining Lin:** Methodology, Investigation, Formal analysis. **Lianjie Peng:** Writing – original draft, Resources, Project administration. **Yiming Hu:** Resources, Formal analysis, Data curation. **Chaoran Pan:** Writing – original draft. **Mouyuan Sun:** Writing – review & editing, Writing – original draft, Visualization, Validation, Supervision, Software, Resources, Project administration, Methodology, Investigation, Funding acquisition, Formal analysis, Data curation, Conceptualization. **Yan Zhang:** Writing – review & editing, Writing – original draft, Visualization, Validation, Supervision, Resources, Investigation, Funding acquisition, Formal analysis, Conceptualization.

## Declaration of competing interest

The authors declare that they have no known competing financial interests or personal relationships that could have appeared to influence the work reported in this paper.
