# Supplementary figures and images for: Ferroptosis-related lncRNAs: Distinguishing heterogeneity of the tumour microenvironment and predicting immunotherapy response in bladder cancer
Source: Heliyon. 2024 May 31;10(11):e32018. doi: 10.1016/j.heliyon.2024.e32018 (PMC11168393; doi:10.1016/j.heliyon.2024.e32018)

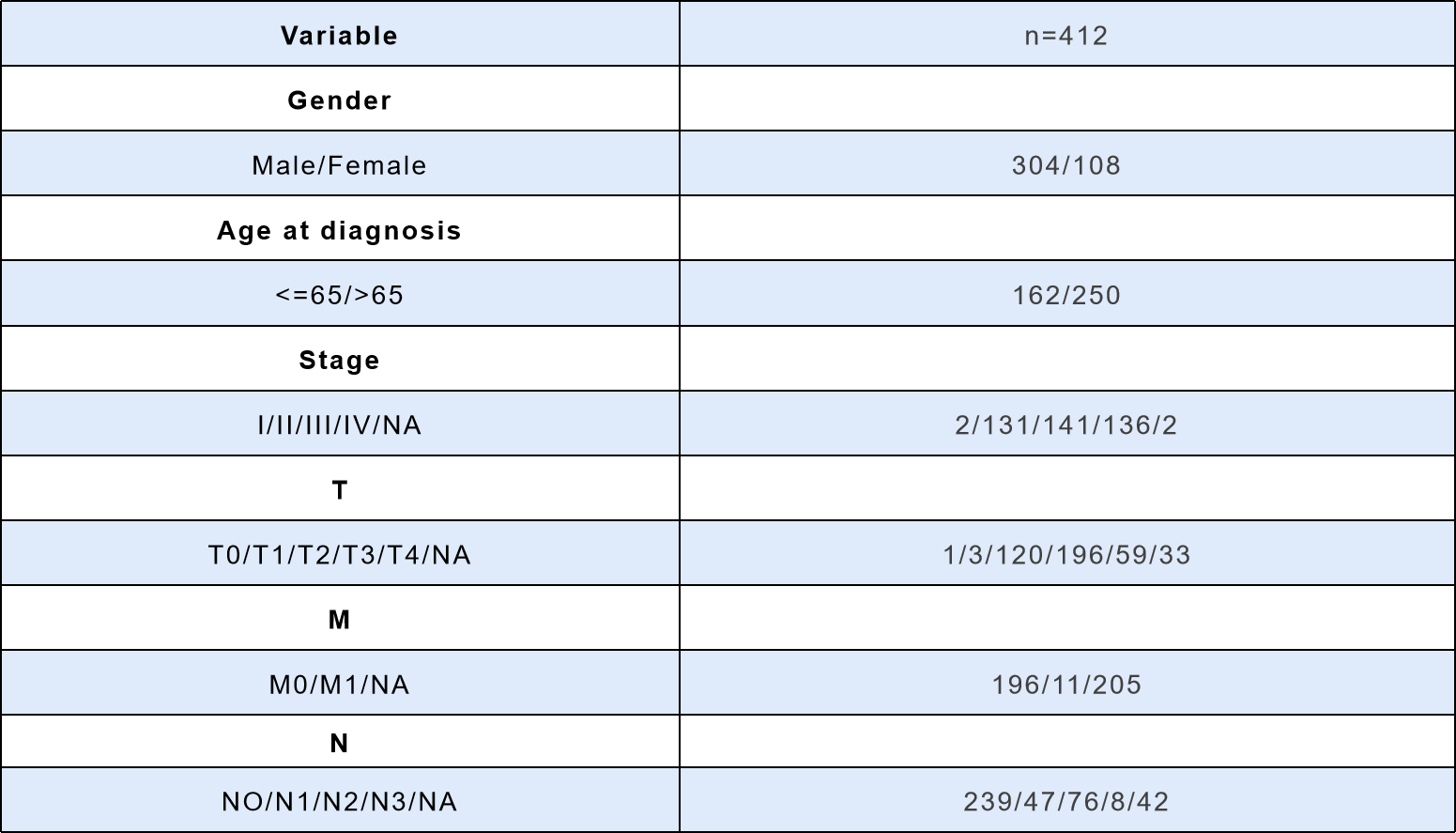


tableS1: Baseline clinical characteristics of BLCA patients

Supplement: Multimedia component 2 [file mmc2.docx]
