# Supplementary material for: Ferroptosis-related lncRNAs: Distinguishing heterogeneity of the tumour microenvironment and predicting immunotherapy response in bladder cancer
Source: Heliyon. 2024 May 31;10(11):e32018. doi: 10.1016/j.heliyon.2024.e32018 (PMC11168393; doi:10.1016/j.heliyon.2024.e32018)
Supplement: Multimedia component 3 [file mmc3.docx]

Gene

ABCC1

ACO1

ACSF2

ACSL3

ACSL4

ACVR1B

AGPAT3

AIFM2

AKR1C1

AKR1C2

AKR1C3

ALB

ALOX12

ALOX12B

ALOX15

ALOX15B

ALOX5

ALOXE3

ANGPTL7

ANO6

ARNTL

ARRDC3

ASNS

ATF3

ATF4

ATG13

ATG16L1

ATG3

ATG4D

ATG5

ATG7

ATM

ATP5MC3

ATP6V1G2

AURKA

BACH1

BAP1

BECN1

BID

BLOC1S5-TXNDC5

BNIP3

BRD4

CA9

CAPG

CARS1

CAV1

CBS

CD44

CDKN1A

CDKN2A

CDO1

CEBPG

CHAC1

CHMP5

CHMP6

CISD1

CISD2

CS

CXCL2

CYBB

DDIT3

DDIT4

DNAJB6

DPP4

DRD4

DRD5

DUOX1

DUOX2

DUSP1

EGFR

EGLN2

EIF2AK4

EIF2S1

ELAVL1

EMC2

ENPP2

EPAS1

FADS2

FANCD2

FBXW7

FH

FLT3

FTH1

FTL

FTMT

Fer1HCH

G6PD

G6PDX

GABARAPL1

GABARAPL2

GABPB1

GCH1

GCLC

GDF15

GLS2

GLUT13

GOT1

GPT2

GPX2

GPX4

HAMP

HBA1

HELLS

HERPUD1

HIC1

HIF1A

HILPDA

HMGB1

HMOX1

HNF4A

HRAS

HSD17B11

HSF1

HSPA5

HSPB1

IDH1

IFNG

IL33

IL6

IREB2

ISCU

JDP2

JUN

KEAP1

KIM-1

KLHL24

KRAS

LAMP2

LINC00336

LINC00472

LOC284561

LOC390705

LONP1

LPCAT3

LPIN1

LURAP1L

MAFG

MAP1LC3A

MAP3K5

MAPK1

MAPK14

MAPK3

MAPK8

MAPK9

MIOX

MIR137

MIR17

MIR212

MIR30B

MIR4715

MIR6852

MIR9-1

MIR9-2

MIR9-3

MT1G

MT3

MTDH

MTOR

MUC1

MYB

NCF2

NCOA4

NF2

NFE2L2

NFS1

NGB

NNMT

NOS2

NOX1

NOX3

NOX4

NOX5

NQO1

NRAS

OTUB1

OXSR1

PANX1

PCK2

PEBP1

PGD

PHKG2

PIK3CA

PLIN2

PLIN4

PML

PRDX1

PRDX6

PRKAA1

PRKAA2

PROM2

PSAT1

PTGS2

RB1

RELA

RGS4

RIPK1

RPL8

RRM2

SAT1

SCD

SCP2

SELENOS

SESN2

SETD1B

SIRT1

SLC1A4

SLC1A5

SLC2A1

SLC2A12

SLC2A14

SLC2A3

SLC2A6

SLC2A8

SLC38A1

SLC3A2

SLC40A1

SLC7A11

SLC7A5

SNORA16A

SNX4

SOCS1

SP1

SQSTM1

SRC

SRXN1

STAT3

STEAP3

STMN1

TAZ

TF

TFAP2C

TFR2

TFRC

TGFBR1

TLR4

TMBIM4

TNFAIP3

TP53

TP63

TRIB3

TSC22D3

TUBE1

TXNIP

TXNRD1

UBC

ULK1

ULK2

VDAC2

VEGFA

VLDLR

WIPI1

WIPI2

XBP1

YWHAE

YY1AP1

ZEB1

ZFP36

ZFP69B

ZNF419

tableS2: List of ferroptosis-related genes
