# Supplementary material for: Ferroptosis-related lncRNAs: Distinguishing heterogeneity of the tumour microenvironment and predicting immunotherapy response in bladder cancer
Source: Heliyon. 2024 May 31;10(11):e32018. doi: 10.1016/j.heliyon.2024.e32018 (PMC11168393; doi:10.1016/j.heliyon.2024.e32018)
Supplement: Multimedia component 1 [file mmc1.docx]

**Ferroptosis-related lncRNAs: distinguishing heterogeneity of tumour microenvironment and predicting immunotherapy response in bladder cancer**

Zhan Yang ^1,+,^ Xiaoqi Li ^1,+^, Lijun Zhou ^1,+^, Yaxian Luo ^2^, Ning Zhan ^2^, Yifan Ye ^1^, Zhichao Liu ^2^, Xiaoting Zhang ^2^, Tao Qiu ^2^, Lining Lin ^2^, Lianjie Peng ^2^, Yiming Hu ^1^, Chaoran Pan ^1^, Mouyuan Sun ^2,*^ & Yan Zhang ^1,*^

1. Department of Urology, The First Affiliated Hospital of Wenzhou Medical University, Wenzhou 325000, China.

2. Stomatology Hospital, School of Stomatology, Zhejiang University School of Medicine, Zhejiang Provincial Clinical Research Center for Oral Diseases, Key Laboratory of Oral Biomedical Research of Zhejiang Province, Cancer Center of Zhejiang University, Engineering Research Center of Oral Biomaterials and Devices of Zhejiang Province, Hangzhou 310000, China.

* Corresponding author.

^+^	These authors contributed equally to this work.

**Figure S1. The flow chart of this study**

**Figure S2. Correlation analysis of 8 central FRGs**

**Figure S3. Screening the key FRLRs and revealing the prognostic significance of these key FRLRs**

**(A-B)** Cvfit and lambda curves showing the LASSO regression were performed with the minimum criteria. **(C-L)** Kaplan-Meier curve to show the overall survival based on AC006106.1, AC021321.1, AC099850.4, AL133415.1, AL583785.1, AL662844.4, AL731567.1, HMGA2-AS1, RBMS3-AS3 and SPAG5-AS1 expression.

**Figure S4. Indicating the contribution of the 10 key FRLRs in BLCA pathogenesis**

**(A-J)** The receiver operating characteristic (ROC) curves based on AC006106.1, AC021321.1, AC099850.4, AL133415.1, AL583785.1, AL662844.4, AL731567.1, HMGA2-AS1, RBMS3-AS3 and SPAG5-AS1 expression.

**Figure S5. The correlation between the stromal score, immune score, estimate score and the 10 key FRLRs(AC006106.1, AC021321.1, AC099850.4, AL133415.1, AL583785.1, AL662844.4, AL731567.1, HMGA2-AS1, RBMS3-AS3 and SPAG5-AS1)**

 **Figure S6. The correlation between the RNAss stemness score (RNAss) , the DNA stemness score (DNAss) and the 10 key FRLRs(AC006106.1, AC021321.1, AC099850.4, AL133415.1, AL583785.1, AL662844.4, AL731567.1, HMGA2-AS1, RBMS3-AS3 and SPAG5-AS1)**

 **Figure S7. Different expression levels of risk score based on clinical characteristics**

**(A)** Different expression levels of risk score between the <=65 and >65 groups. **(B)** Different expression levels of risk score between the female and male groups. **(C)** Different expression levels of risk score between the stageⅠ-Ⅱ and stage Ⅲ-Ⅳ groups. **(D)** Different expression levels of risk score between the T0-2 and T3-4 groups. **(E)** Different expression levels of risk score between the M0 and M1 groups. **(F)** Different expression levels of risk score between the N0 and N1-3 groups.

**Figure S8. Functional enrichment analysis of differentially expressed gene sets and pathways between high- and low-risk groups.**

**(A)** Visualizations of Gene ontology (GO) analysis in the training cohort. **(B)** Kyoto encyclopedia of genes and genomes (KEGG) analysis of the training cohort. **(C)** Visualizations of Gene ontology (GO) analysis in the training cohort. **(D)** Kyoto encyclopedia of genes and genomes (KEGG) analysis of the training cohort.

**Figure S9. Comparison of tumour microenvironment related genes and multiple score between the high- and low-risk groups in the TCGA cohort.**

**(A)** Different expression levels of TGF beta- and EMT pathway-related genes between the high- and low-risk groups. **(B)** Different expression levels of immune-activated genes between the high- and low-risk groups. **(C)** The EstimateScore between the high- and low-risk groups. **(D)** The ImmuneScore between the high- and low-risk groups. **(E)** The StromalScore between the high- and low-risk groups.

 **Figure S10. Different expression levels of HMGA2-AS1, AC099850.4 and AL662844.4 between different tumour stages and grades**

**(A-C)** Different expression levels of HMGA2-AS1, AC099850.4 and AL662844.4 between the StageⅠ-Ⅱ and Stage Ⅲ-Ⅳ groups. **(D-F)** Different expression levels of HMGA2-AS1, AC099850.4 and AL662844.4 between the hige grade and low grade groups.

**Figure S11. Investigation of the biological pathways and mechanisms regulated by ESM1.**

**(A)** Volcano plot of DEGs in the high- and low- ESM1 expression groups; the red point indicated the gene with a log2FC > 1 and Log2FC< -1, while the blue point indicated the opposite. **(B-C)** GO and KEGG enrichment analysis of the DEGs. **(D)** The top six most significant pathways by GSEA in the high- and low- ESM1 expression groups.

**Figure S12. Drug sensitivity and immunotherapy analysis of the ceRNA network**

**(A)** Kaplan-Meier curve；The expression levels of NEGR1；The expression levels of NEGR1 among different tumour stages; ROC curves of anti-PD- 1, anti-PD-L1 and anti-PD- 1/CTLA-4 based on NEGR1 expression; Drug sensitivity analysis. **(B)** Kaplan-Meier curve；The expression levels of JAZF1；The expression levels of JAZF1 among different tumour stages; ROC curves of anti-PD- 1 and anti-CTLA-4 based on JAZF1 expression; Drug sensitivity analysis. **(C)** Kaplan-Meier curve；The expression levels of JCAD；The expression levels of JCAD among different tumour stages; ROC curves of anti-PD- 1/PD-L1, anti-PD-L1 and anti-PD- 1/CTLA-4 based on JCAD expression; Drug sensitivity analysis. **(D)** Kaplan-Meier curve；The expression levels of NFIB；The expression levels of NFIB among different tumour stages; ROC curves of anti-PD- 1, anti-PD-L1 and anti-PD- 1/PD-L1 based on NFIB expression; Drug sensitivity analysis.
